# Antibody-Drug Conjugates for the Treatment of HER2-Positive Breast Cancer

**DOI:** 10.3390/genes13112065

**Published:** 2022-11-08

**Authors:** Mariana K. Najjar, Sara G. Manore, Angelina T. Regua, Hui-Wen Lo

**Affiliations:** 1 Wake Forest Graduate School of Biomedical Sciences, Wake Forest University School of Medicine, Winston-Salem, NC 27101, USA; 2 Department of Neurosurgery, McGovern Medical School, University of Texas Health Science Center at Houston, MSE R162, 6431 Fannin Street, Houston, TX 77030, USA; 3 Department of Cancer Biology, Wake Forest University School of Medicine, Winston-Salem, NC 27101, USA

**Keywords:** HER2, antibody drug conjugate, ADC, T-DM1, Enhertu, trastuzumab, monoclonal antibody, breast cancer, therapeutics, cancer

## Abstract

Human epidermal growth factor receptor 2 (HER2) receptor tyrosine kinase is overexpressed in 20–30% of breast cancers and is associated with poor prognosis and worse overall patient survival. Most women with HER2-positive breast cancer receive neoadjuvant chemotherapy plus HER2-targeted therapies. The development of HER2-directed therapeutics is an important advancement in targeting invasive breast cancer. Despite the efficacy of anti-HER2 monoclonal antibodies, they are still being combined with adjuvant chemotherapy to improve overall patient outcomes. Recently, significant progress has been made towards the development of a class of therapeutics known as antibody-drug conjugates (ADCs), which leverage the high specificity of HER2-targeted monoclonal antibodies with the potent cytotoxic effects of various small molecules, such as tubulin inhibitors and topoisomerase inhibitors. To date, two HER2-targeting ADCs have been approved by the FDA for the treatment of HER2-positive breast cancer: Ado-trastuzumab emtansine (T-DM1; Kadcyla^®^) and fam-trastuzumab deruxtecan-nxki (T-Dxd; Enhertu^®^). Kadcyla and Enhertu are approved for use as a second-line treatment after trastuzumab-taxane-based therapy in patients with HER2-positive breast cancer. The success of ADCs in the treatment of HER2-positive breast cancer provides novel therapeutic advancements in the management of the disease. In this review, we discuss the basic biology of HER2, its downstream signaling pathways, currently available anti-HER2 therapeutic modalities and their mechanisms of action, and the latest clinical and safety characteristics of ADCs used for the treatment of HER2-positive breast cancer.

## 1. Introduction

Breast cancer is the most common cancer among American women and is the second leading cause of cancer-related deaths in women worldwide [1]. Breast cancer is a heterogeneous disease that can be classified into four molecular subtypes based on cell-surface receptor expression: Luminal A, Luminal B, human epidermal growth factor receptor 2 (HER2)-positive, and triple-negative breast cancer (TNBC); each of these breast cancer subtypes has distinct characteristics, epidemiology, responses to therapy, and prognoses [2,3,4]. Luminal A and B subtypes of breast cancer are the most prevalent subtypes. Luminal A breast cancer expresses both estrogen receptors (ER) and progesterone receptors (PR), while Luminal B tumors expresses ER but may or may not express PR [4,5,6,7]. In addition to hormone receptors, Luminal B breast cancer may also express HER2 receptors [5,6,8]. HER2-positive breast cancers are mainly characterized by overexpression of HER2 and they are considered the second most aggressive subtype [3,4]. TNBC does not express either ER, PR or HER2; it is considered to be the most aggressive subtype of breast cancer [3,4,8].

Approximately 20–30% of breast cancer patients have an amplification and/or overexpression of HER2, which is associated with poor prognosis and short overall survival [9,10,11]. Thus, HER2 became an optimal target in the therapeutic intervention and management of HER2-positive breast cancers, and in the development of targeted therapeutics. In the early to mid-1990s, a humanized monoclonal antibody (mAb), trastuzumab, was developed to directly bind to the HER2 protein preventing downstream signaling [12,13,14]. Due to the overwhelming response in HER2-positive breast cancer patients, trastuzumab received FDA approval for the treatment of HER2-positive breast cancer as an adjuvant first line therapy in 1998 [15]. The development of trastuzumab remains one of the most significant advancements in the treatment of HER2-positive breast cancer and dramatically influenced the therapeutic modalities for patients with HER2-positive breast cancer. Subsequently, a variety of HER2-targeting agents including mAB pertuzumab and small molecule inhibitors, such as lapatinib and neratinib, were later approved for the treatment of HER2-positive breast cancer [16,17,18]. Overall, HER2-targeting agents are associated with improved response rate (RR), progression free survival (PFS), and overall survival (OS) [19,20,21].

Despite the availability of HER2-targeting agents, approximately 5.8–8.6% of HER2-positive patients relapse due to acquired therapeutic resistance to anti-HER2 mAb [22,23,24]. In order to overcome this therapeutic resistance and to achieve maximal antitumoral activity, anti-HER2 mAbs are being augmented with taxane-based cytotoxic chemotherapies creating the foundation for a series of smart chemotherapeutics now known as antibody-drug conjugates or ADCs. ADCs combine the tumor specificity of mAb and the cytotoxicity of small molecule chemotherapeutics into one single pharmaceutical entity. The development process of ADCs is long and complex, extending the period from the proposal of the ADC design to the approval of the first ADC to over 100 years [25,26]. ADCs have created a new era of targeted therapy that greatly improves the prognosis of breast cancer patients [27,28,29,30,31]. Two anti-HER2 ADCs, ado-trastuzumab emtansine (T-DM1; Kadcyla^®^) and fam-trastuzumab deruxtecan-nxki (T-DXd; Enhertu^®^) are approved as adjuvant therapies and rescue treatments for patients with HER2-positive breast cancer, and most recently HER2-low breast cancer [32,33,34]. To date, several ongoing clinical trials assess efficacy of ADCs in the treatment of breast cancer and other solid tumors (discussed later), highlighting the overall potential of ADCs as promising treatment options against cancer [35]. This review covers the basic biology behind the structure and mechanism of action of ADCs, summarizes the advantages ADCs have in overcoming therapeutic resistance, discusses the metabolic profile of ADCs, lists several ADCs currently under development, and includes up-to-date in-depth information on FDA-approved ADCs for the treatment of HER2-positive and, most recently, HER2-low breast cancer along with their toxicity profiles and current ongoing clinical trials.

## 2. HER2 and HER2-Targeted Therapy in Breast Cancer

HER-2 is a member of the epidermal growth factor receptor (EGFR) family of receptor tyrosine kinases comprised of EGFR/HER1, HER2, HER3, and HER4. EGFR family of receptors play pivotal roles in normal cell growth and differentiation [36,37,38]. However, overexpression or abnormal activation of these receptors is linked to the pathogenesis of several human cancers including breast, ovarian and gastric [39,40,41]. The HER2 receptor is a 1255 aa, 185-kDa transmembrane protein whose locus is located on chromosome 17q21.1 [11,42,43,44]. HER2 is expressed in many normal tissues at low levels, and its overexpression was linked to excessive/unrestrained cell growth and proliferation leading to tumorigenesis [10,11,22,36,37,38,45,46]. In breast cancer, the *HER2* gene is amplified in 20–30% of primary tumors; this amplification and resulting overexpression of HER2 protein are correlated with enhanced activity in signaling pathways involved in potent proliferative and antiapoptotic signals [10,11]. HER2 amplification and/or overexpression promotes aggressive disease phenotypes characterized by high resistance rate and shortened survival rates [10].

### 2.1. HER2 Activation

**Figure 1:**
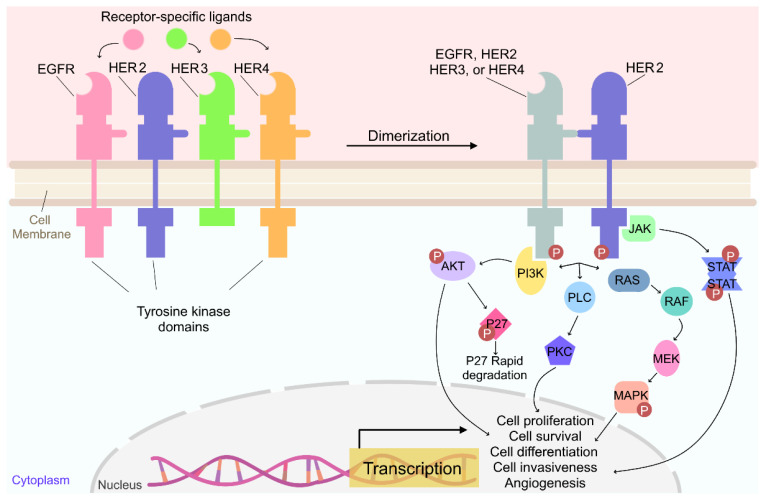
**Overview of the HER2 signaling pathway.** Unlike the other EGFR family of receptors, HER2 does not bind to any known ligands. Instead, HER2 is activated following heterodimerization with other activated EGFR family of receptors or by heterodimerization with activated HER2 receptors. Receptor dimerization leads to the phosphorylation of tyrosine residues and resultant signal transduction. PI3K/AKT, RAS/MEK/MAPK, JAK/STAT, and PKC are the most common signaling pathways through which several downstream cascades are activated, promoting numerous effects, including cell proliferation, survival, differentiation, angiogenesis, and invasion. Moreover, activated PI3K/AKT also leads to the degradation of cell-cycle inhibitor p27^Kip1^ and thus promotes cell-cycle progression. EGF, Epidermal growth factor; HB-EGF, heparin-binding epidermal growth factor; TGF, tumor growth factor; NRG, neuregulin.

Members of the EGFR family of receptors exist as monomers, when inactive, on the surface of cells and their structures are primarily comprised of three domains: a cysteine-rich extracellular domain, a lipophilic transmembrane segment, and an intracellular tyrosine kinase domain [47,48]. With the exception of HER2 that does not bind to any ligands, the EGFR family of receptors are mainly activated through ligand binding to the extracellular domain, which promotes subsequent receptor dimerization, autophosphorylation, and transphosphorylation of their intracellular tyrosine kinase domains (Figure 1) [49,50,51,52]. In the absence of ligand-induced dimerization/activation, HER2 becomes activated when it heterodimerizes with EGFR, HER3, or HER4, or homodimerizes with another HER2 monomer. HER2 activation leads to subsequent activation of downstream signaling pathways such as PI3K/AKT, RAS/MEK/MAPK, JAK/STAT, and PKC [49,50,51,52]. Studies have demonstrated that the most active and most potent tumor-enhancing effect is achieved through the HER2/HER3 dimer, which functions mostly through the downstream activation of PI3K/AKT, MAPK/ERK and JAK/STAT pathways and is responsible for treatment failure and increased resistance to therapies in breast cancer patients [53,54,55]. Besides members of the EGFR family, HER2 dimerize with other membrane-bound receptors, such as insulin-like growth factor 1 (IGF-1), leading to an increase in the phosphorylation of HER2 and consequent activation of tumor-promoting downstream signaling pathways [56].

HER2 hetero- and homo-dimerization leads to auto- and trans-transphosphorylation of tyrosine residues located on the intracellular domain which, in turn, facilitates docking of various other intracellular proteins and can elicit activation of downstream signaling pathways [49,50,51,52]. Y1005, Y1023, Y1139, Y1196, Y1222, and Y1248 are among the main tyrosine residues that are readily phosphorylated and are important for HER2 kinase activity [57,58,59,60,61,62]. HER2 activation promotes the activation of transcription factors that regulate genes involved in cell proliferation, survival, differentiation, angiogenesis, and invasion [36,49,63]. Moreover, dimerization of the HER2 receptor leads to mislocalization and degradation of the cell-cycle inhibitor p27^Kip1^ and thus promotes cell-cycle progression [45] (Figure 1).

### 2.2. HER2 Overexpression in Breast Cancer

Activating *HER2* mutations and *HER2* amplification are early events in breast tumorigenesis occurring in almost 50% of in situ carcinomas and, in 20% of the cases, are maintained during progression of the disease to the invasive type [36,64]. Multiple studies revealed that amplification of the *HER2* gene is associated with higher cancer recurrence rate and shorter disease-free and overall survival [46,65]. Moreover, *HER2*-amplified breast cancers display increased sensitivity to certain chemotherapeutic agents, such as doxorubicin, increased resistance to certain hormonal agents, such as tamoxifen, and increased propensity to metastasize to the brain, lungs, and liver [66,67]. These findings highlight the significance of HER2 as a prognostic marker, and the predictive implications HER2 has in breast cancer [11].

Several methods have been developed for the identification of the HER2 status but only two methods, immunohistochemistry (IHC) and fluorescence in situ hybridization (FISH), are currently approved by the American Society of Clinical Oncology (ASCO) and the College of American Pathologists (CAP) for assessment of HER2 status [68,69,70]. All patients with invasive breast cancer are required to have their HER2 status determined based on one or more test results [68,69,70]. Patient derived specimens must undergo an initial HER2 testing by IHC assay (giving a score of 0 to 3+) followed by a validation through FISH (giving positive or negative results) [68,69,70]. Generally, only specimens that test IHC 3+ or FISH positive respond to anti-HER2 treatments; an IHC 2+ test result is called borderline and is usually validated with a FISH test [68,69,70].

### 2.3. Targeting HER2

Ever since the clinical relevance of HER2 in breast cancer was discovered and understood, HER2 became an optimal therapeutic target in a large subset of patients harboring *HER2* gene amplification and protein overexpression. A variety of anti-HER2 agents including, trastuzumab, pertuzumab, lapatinib, and neratinib have been approved by the FDA for the treatment of HER2-positive breast cancer [16,17,18,71]. Trastuzumab, developed in 1990, is the first HER2-targeting mAb approved by the FDA for the treatment of HER2-positive breast cancer [13]. Trastuzumab (Herceptin^®^) binds to the dimerization domain of HER2 and inhibits homodimerization, thereby preventing HER2 activation and inhibiting downstream signaling [72]. Several mechanisms of trastuzumab actions have been proposed including, inhibition of HER2 shedding, inhibition of PI3K/AKT pathway, antibody-dependent cellular cytotoxicity, and inhibition of tumor angiogenesis (Table 1). Pertuzumab (Perjeta^®^), which received FDA approval in 2017, is another HER2-targeting mAB which elicits its anti-HER2 activity via a slightly different mechanism: it binds to the dimerization domain and inhibits ligand-induced heterodimerization between HER2 and HER3, leading to reduced signaling via intracellular pathways [20,73]. Lapatinib (Tykerb^®^) and neratinib (Nerlynx^®^) are small molecule inhibitors that inhibit HER2 tyrosine kinase activity [74,75]. Lapatinib and neratinib both directly bind to the intracellular tyrosine kinase domains and inhibit kinase activity preventing the activation of downstream signals [74,75,76].

### 2.4. Resistance to Anti-HER2 Therapies

Despite the targeted specificity of the anti-HER2 agents described, breast cancer patients continue to acquire therapeutic resistance with prolonged treatment [86,87]. The main mechanisms of resistance to anti-HER2 agents are (1) overabundance of HER ligands and receptors allowing for alternative dimerization that lead downstream pathways to continue to signal despite being partially inhibited [87]; (2) reactivation of pathway signaling through loss of downstream negative-regulators, or gain of activating mutations [79,88,89]; (3) employment of alternative pathways to escape HER2 blockade and to continue drive the growth of cancer cells [90,91]. Since the development of resistance to HER2-targeted therapy is common amongst HER2-positive breast cancer patients, it has prompted investigation into use of a combinatorial therapeutic regimen that combines anti-HER2 mAb with chemotherapeutic agents. Investigation into these novel anti-HER2 combinatorial treatment modalities has led to the emergence of a new monoclonal antibody technology known as ADCs. With the proper drug/linker design and mode of internalization, ADCs present with features that have a potential for overcoming drug resistance and improving therapeutic outcomes for patients with breast cancer [92].

## 3. Antibody-Drug Conjugates for HER2-Positive Breast Cancer

In 2000, the first ADC was approved by the FDA for the treatment of acute myeloid lymphoma [26]. Given the prognostic value of HER2 in breast cancer, HER2 is regarded as a potent therapeutic target for HER2-positive breast cancers. To date, nearly thirty HER2-targeting ADCs have been developed among which twenty-three positive results in clinical trials, and two of these ADCs have received FDA approval in the United States (Table 2).

### 3.1. Composition of Anti-HER2 ADCs

**Figure 2:**
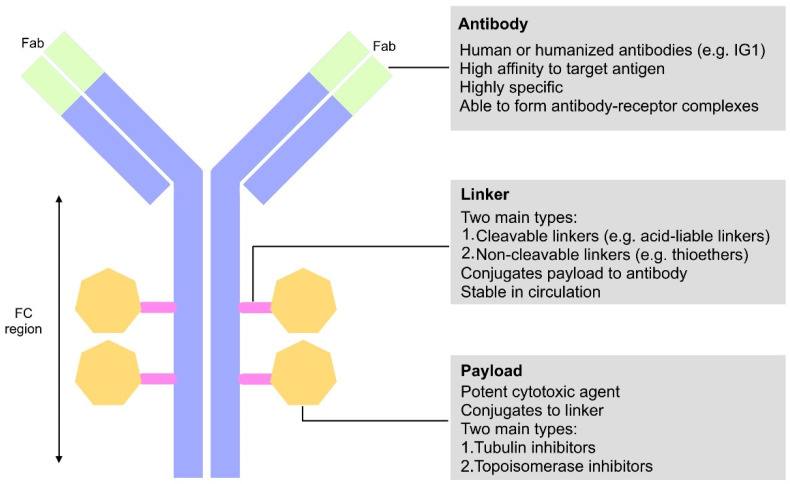
**Schematic diagram of an ADC showing the general structure and favorable characteristics.** The antibody contains antigen-binding sites (F_ab_) engineered to recognize target antigens. Payloads are connected to the antibody via linkers.

ADCs consist of a humanized mAb, mainly immunoglobulin G (IgG), linked to a small molecular cytotoxic agent, known as the payload, using a cleavable or non-cleavable molecular linker (Figure 2) [114]. However, given the complexity of the anti-tumor mechanism of ADCs, several critical requirements in their structures have been highlighted for the development of a treatment with the desired efficacy while eliminating or reducing high grade adverse events (AEs) [114].

#### 3.1.1. Target Antigen

The design of an ADC revolves around the target antigen. Ideally, the target antigen should be tumor specific and preferentially expressed in tumor tissues but lowly expressed in non-tumorigenic tissues. Moreover, the target antigen must be easily accessible to antibody binding to facilitate effective internalization and delivery of the active cytotoxic drug into the tumor cell [115,116]. Currently, two antigens are approved for targeting breast cancer with ADCs, HER2 and TROP2. HER2 is overexpressed in 15–20% of breast cancer while TROP2 is overexpressed in more than 85% of triple negative breast cancer (TNBC) [117,118]. The potential use of other target antigens, such as EGFR and Notch3, in the development of new ADCs is currently being investigated [119,120,121].

#### 3.1.2. Antibody

The compatibility and specificity of the antibody is critical for the activity of an ADC. The mechanism of action of ADCs relies on the internalization of antibody-bound antigens for the delivery of cytotoxic agents [115,116]. Hence, the antibody must have a high affinity to the target antigen to achieve maximum effect while reducing cross-reactivity that may reduce efficacy of the cytotoxic component of the ADC [122]. In addition, to prevent production of anti-drug antibodies (ADAs) by the immune system, it is critical that the antibody component of the ADC has reduced immunogenicity [115,116], which is achieved through the use of humanized antibodies. All ADCs approved for the treatment of HER2-positive breast cancer utilize humanized immunoglobulin G1 (IgG1) as a monoclonal antibody for targeting HER2 [35,115,116].

#### 3.1.3. Linker

Linkers within the ADC function to conjugate the monoclonal antibody with the cytotoxic payload. Linkers should be highly stable in the blood circulation to allow the release and internalization of the ADC, though only upon antibody binding with the antigen. Unstable linkers may release the cytotoxic drug prematurely before reaching the tumor, hence, reducing the efficacy of the ADC treatment and increasing chances of off-target toxicity [35].

A linker can be classified as either cleavable or non-cleavable depending on its composition and susceptibility to proteolytic degradation within the cell. Non-cleavable linkers are more stable in circulation [115]; however, after proteolytic degradation, charged amino acid residues may be retained on the cytotoxic payload and can interfere with the overall efficacy of the drug [115]. The most commonly used type of non-cleavable linkers in ADCs is thioether linkers, currently used in T-DM1 [123]. Cleavable linkers depend on the physiological conditions of the cell and can be subdivided into two types: pH-sensitive and protease-cleavable linkers [35,115]. pH-sensitive linkers utilize the lower pH in the endosomes and lysosomes of target tumor cells to trigger hydrolysis of acid labile groups within a linker, while protease-cleavable linkers utilize common proteases, found in lysosomes of target tumor cells, for specific peptide sequence recognition and cleavage in the linker [35,115,124]. It is important to note, however, that cleavable linkers display nonspecific release of the cytotoxic drug [35,115]. The most commonly used cleavable linkers include acid-labile hydrazones, or disulfides [35,115].

#### 3.1.4. Payload

Cytotoxic payloads selected for ADCs are usually highly potent and extremely cytotoxic agents; hence, their development as free drugs is often clinically limited [125,126,127,128]. Ideally, ADCs allow for stable transfer of cytotoxic agents in the circulation while resisting off-target release. Conjugation of the payload to the linker is critical for the efficacy of an ADC and should be made possible by the chemical structure of the chosen cytotoxic agent [125]. In addition, the therapeutic entity of choice must be highly potent to elicit the desired therapeutic efficacy. Several early ADCs that use standard chemotherapeutics as the payload demonstrated preclinical efficacy but failed in the clinical setting [129,130,131]. This poor clinical response to ADCs was mainly due to the suboptimal therapeutic efficacy within the tumor [132].

The number of payloads an ADC can carry, also known as drug-to-antibody ratio (DAR) is limited and can range from 0–8 payload molecules [116]. Alternatively, the number of antigens ADCs bind and deliver to are limited, thus it is necessary to choose cytotoxic agents that have a low half-maximal inhibitory concentration (IC50) to achieve optimal therapeutic concentration while reducing the potential off-target effects to surrounding normal tissues [116]. Currently, the most used drug classes in ADCs are tubulin inhibitors and topoisomerase inhibitors [116,133].

### 3.2. Mechanism of Action of Anti-HER2 ADCs

**Figure 3:**
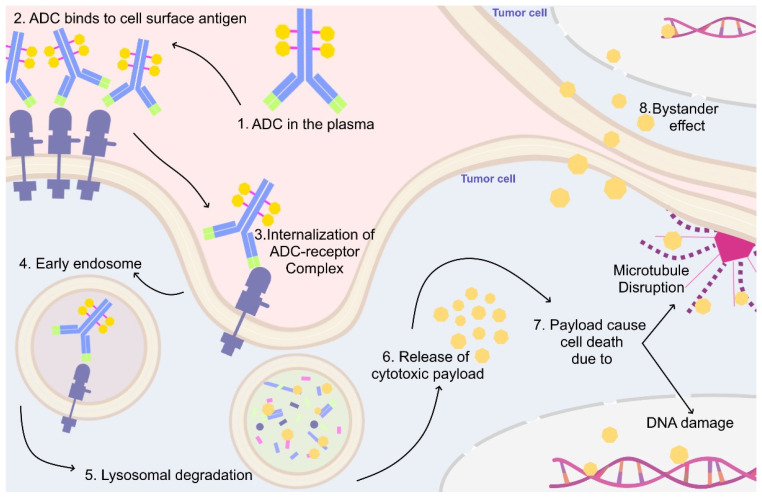
**Antibody-drug conjugate mechanism of action.** Once the ADC is administered, (1) it is released into the bloodstream. (2) The antibody portion of the ADC binds to overexpressed target tumor antigen/receptor (e.g., HER2). (3) Upon binding, the ADC-receptor complex undergoes receptor-mediated endocytosis, leading to the formation of endosomes (4). Within the lysosome, (5) the ADC-receptor complex is degraded, and the linker is cleaved, leading to the release of cytotoxic payloads (6). Depending on the type of payload used, (7) it will cause cell death either through DNA damage or microtubule disruption. Additionally, (8) payloads that have a membrane-permeable nature will exert the same cytotoxic effect on neighboring cells through a process known as the bystander effect, regardless of their antigen expression.

Anti-HER2 ADCs share one main mechanism for targeting tumors where mABs function as transport systems carrying cytotoxic payloads to HER2 overexpressing tumor cells and binding to the extracellular domain of the HER2 protein [134,135,136]. Once bound, antibodies linked with cleavable linkers release the payloads into or around tumor cells, whereas antibodies linked with non-cleavable linkers are internalized by the tumor cells and depend on lysosomal degradation to release the payload [134,135,136]. Depending on their structures, payloads can be membrane permeable and can affect surrounding tumor cells regardless of their HER2 expression, a phenomenon known as the bystander effect [137] (Figure 3). The bystander effect characteristic allows for increased bioavailability of cytotoxic payload and improves the efficacy of the drug in heterogenous tumors [137].

### 3.3. Metabolism of Anti-HER2 ADCs

Anti-HER2 ADCs share a common structural construct designed to stably travel through the bloodstream and selectively deliver cytotoxic payloads to HER2-expressing cells [134,135,136]. To achieve optimal drug delivery while reducing off-target drug release, a number of elements pertaining each component of the ADC structure need to be optimized [138,139,140,141,142,143]. The mAb portion of anti-HER2 ADCs is distinctly metabolized by proteolytic degradation within the cancer cells; changes in the binding-regions of the mAb impact the ADC/cancer cell interaction and can result in altered metabolism [144]. In addition to the structure of the mAb, the type of linkers selected plays an important role in the metabolism of the ADC [138,144]. A 2-fold increase in ADC clearance was reported with the use of a disulfide-linked T-DM1 when compared to the thioether-linked T-DM1 [138]. Moreover, the stability of ADCs in the bloodstream is also found to be linker dependent [140,141,144]. ADCs are reported to have shorter terminal half-lives compared to their respective unconjugated mAb [140,141]. The number of cytotoxic payloads conjugated to the mAb also affects the metabolism of the ADC [139,144]. ADCs with a higher drug-antibody ratio (DAR) tend to have increased clearance and reduced drug exposure [139]. Additionally, the type of cytotoxic payloads used in the ADC construct affect the metabolic profile of the ADC [142,143,144]. After the administration of ADCs, off-target release of cytotoxic payloads may lead to metabolism by cytochrome P450 enzymes [142,143]. Given the potency of cytotoxic payloads, changes in their exposure due to P450 enzymes-mediated drug-drug interactions (DDIs) may affect the safety profile of the ADC in patients [142,143]. Thus, evaluations of the metabolism of each structural component should be considered in the development of the ADC.

### 3.4. Current FDA Approved ADCs for HER2-Positive Breast Cancer

#### 3.4.1. Ado-Trastuzumab Emtansine (Kadcyla^®^)

Ado-trastuzumab emtansine (T-DM1; Kadcyla^®^) was the first anti-HER2 ADC to receive FDA approval [7,19]. In 2013, T-DM1 was approved as a single therapy for the treatment of advanced-stage HER2-positive early breast cancer (EBC) after neoadjuvant treatment with trastuzumab and a taxane (paclitaxel or docetaxel) [19]. More recently, in 2019, the FDA expanded the approved use of ado-trastuzumab emtansine for the treatment of early-stage high-risk HER2-positive breast cancer patients with residual invasive disease after neoadjuvant taxane and trastuzumab-based treatment [96]. T-DM1 is comprised of the monoclonal antibody trastuzumab that islinked to mertansine (DM1), a potent microtubule polymerization inhibitor, via a non-cleavable maleimidomethyl cyclohexane-1-carboxylate (MCC) thioether linker [145,146,147]. The T-DM1 structure retains both the cytotoxic functions of trastuzumab, including cellular cytotoxicity and signal inhibition, and the antitumoral effects of DM1 [145,146,147]. DM1, by itself, is known to have a relatively narrow therapeutic window, but its linkage to trastuzumab, with a DAR of 3.5, increased DM1 targeted selectivity and thereby widened its therapeutic window [148]. The indication for T-DM1 as a second line treatment in advanced-stage HER2-positive breast cancer, and more recently for early-stage patients with invasive residual disease, are based on data from the EMILIA, TH3RESA and KATHERINE phase III clinical trials [19,30,96,149,150] (Table 3).

The initial approval of T-DM1 was based on the EMILIA study. The EMILIA was a phase III clinical trial that investigated the efficacy of T-DM1 vs. capecitabine and lapatinib, the standard second-line therapy at the time, in HER2-positive metastatic breast cancer patients progressing after treatment with trastuzumab and a taxane (n = 991) [19,149]. The results of the study favored T-DM1 which demonstrated an improvement in median overall survival (OS) (30.9 vs. 25.1 months; HR 0.68; 95% CI: 0.55–0.85; *p* < 0.001) and progression free survival (PFS) (9.6 vs. 6.4 months; HR 0.65; 95% CI: 0.55–0.77; *p* < 0.001) after a 47.8-month median follow-up [19]. In this study, most noted adverse events in the T-DM1 cohort were changes in clinical laboratory test results, including thrombocytopenia and elevated serum aminotransferase levels [19]. Moreover, in the lapatinib plus capecitabine cohort, incidences of symptomatic adverse events including diarrhea, nausea, vomiting, and palmar–plantar erythrodysesthesia were higher [149].

Furthermore, the observed superiority of T-DM1 in the EMILIA study was further confirmed in the TH3RESA phase III clinical trial. T-DM1 demonstrated an improvement in OS when compared to physician’s choice of therapy (22.7 vs. 15.8 months; HR 0.552; 95% CI: 0.37–0.83; *p* = 0.0034) and in PFS (6.2 vs. 3.3 months; HR 0.53; 95% CI: 0.42–0.66; *p* < 0.0001) after a 7.2-month median follow-up [30]. Relative to the EMILIA study, the most noted adverse events in the T-DM1 cohort of the TH3RESA study were thrombocytopenia whereas in the physician’s choice therapy, the most frequently observed adverse events were neutropenia, diarrhea, and febrile neutropenia [30].

Recently, the approved use of T-DM1 was extended to include the treatment of early-stage high-risk HER2-positive breast cancer patients with residual invasive disease after neoadjuvant treatment [151]. This expansion in the indication was based on the results of the KATHERINE study [96,151]. The KATHERINE was a phase III clinical trial in which patients showed significant increase in the 3-year invasive disease-free survival (IDFS) in the T-DM1 cohort vs. cohort treated with trastuzumab alone (87.8% vs. 77.8%; HR 0.5; 95% IC: 0.39–0.64; *p* < 0.001) [96]. The safety data of T-DM1 was consistent with both EMILIA and TH3RESA studies where the most reported adverse events were thrombocytopenia [96]. In addition, higher-grade adverse events associated with T-DM1 induced thrombocytopenia were reported when compared with trastuzumab alone [96].

In summary, T-DM1 was found to be associated with manageable symptomatic adverse events, mostly grade 1–2 in severity; including gastrointestinal (GI) toxicity, neuropathy and left ventricular ejection fraction (LVEF) decline [19,30,96]. The most frequently reported high-grade toxicities include thrombocytopenia, which is a dose-limiting toxicity, and increases in liver enzymes leading to potential liver toxicities [19,30,96].

Currently, there is an ongoing study, ATOP phase II clinical trial (NCT03587740), investigating the potential of replacing the use of trastuzumab plus chemotherapy with T-DM1 as a single first line treatment in patients with early HER2-positive breast cancer [70]. Investigation of this approach started in 2013 with a phase II clinical trial named ATEMPT and recently ended producing no definitive results [28]. To date, it is still unknown if T-DM1 is beneficial to patients who have not received neoadjuvant therapy.

Moreover, in a phase II basket trial, T-DM1 was determined to be effective against tumors with HER2 mutations, regardless of amplification or expression status [27]. This was the first positive trial investigating this molecular subset, which resulted in a warranted use of T-DM1 for further studies and trials [30,149,150]

In 2016, a phase Ib/IIa clinical trial was conducted to investigate the combinatorial effects of T-DM1 and docetaxel, and potentially pertuzumab, in patients with HER2-positive advanced breast cancer [152]. The results from the study reported that T-DM1 combined with docetaxel ± pertuzumab appeared efficacious; however, nearly 50% of the patients experienced AEs requiring dose reductions [152]. More recently, a phase II clinical trial, NSABP Foundation Trial FB-10, studied the safety and tolerability of T-DM1 plus neratinib in patients with metastatic HER2-positive breast cancer, and reported a recommended dose for this regimen to provide basis for future studies that would better define the activity of this combination [153].

#### 3.4.2. Fam-Trastuzumab Deruxtecan-Nxki (Enhertu^®^)

Fam-trastuzumab deruxtecan-nxki (DS-8201a; T-DXd; Enhertu^®^) was the second anti-HER2 ADC to receive FDA approval. In 2019, it was approved for HER2-positive unresectable or metastatic breast cancer treated with at least two prior lines of HER2-targeting regimens [95]. Following that, in 2021, the approved use was expanded for the treatment of patients with previously treated HER2-positive advanced gastric cancer [94]. Most recently, in 2022, T-DXd was approved in the US for the treatment of patients with HER2-positive metastatic breast cancer with prior HER2-targeted treatment [154]. Subsequently, T-DXd was approved for the treatment of patients with unresectable, or metastatic HER2-low breast cancer with prior chemotherapy or recurrence [155]. The approved indications of T-DXd are based on data from the phase I & II DESTINY-BREAST01 clinical trial, DESTINY-BREAST03 phase III clinical trial, and the DESTINY-BREAST04 phase III clinical trial [31,34,93,95,154,156] (Table 4).

Like T-DM1, the structure of T-DXd is comprised of the mAb trastuzumab [156]. However, instead of DM1, trastuzumab is linked to a cytotoxic derivative of exatecan, a potent topoisomerase I inhibitor, via a cleavable Maleimide tetrapeptide linker with a DAR of 8 (compared T-DM1, having a DAR of 3.5) [156]. The cleavable linkers are acted upon by lysosomal enzymes, cathepsins, that are vastly available in many cancer cells [157,158]. Once cleaved, and with the membrane permeable property of the payloads, cytotoxic exatecan derivatives are capable of exerting bystander effects and thus achieving improved efficacy in heterogenous tumors [157,158].

Initially, T-DXd received accelerated approval for the treatment of HER2-positive unresectable or metastatic breast cancer following two or more prior anti-HER2 based regimens [159]. This indication was approved after the results of the first in-human phase I clinical trial and the phase II DESTINY-BREAST01 trial [93,95,156]. The phase I clinical trial for T-DXd assessed the safety, tolerability, and activity of the drug against patients with pretreated advanced HER2-positive breast cancer (n = 111) [93]. The study showed that 59.5% (95%CI: 49.7–68.7) of the patients showed favorable objective response with T-DXd monotherapy after a 20.7-month median follow-up. Moreover, 19% of the patients showed at least one serious treatment-emergent adverse event, including anemia and/or decreased neutrophil, white blood cell, and platelet counts [93]. The results of the phase I clinical trial led to the registration of a phase II trial, DESTINY-BREAST01 [95]. It investigated the effect of T-DXd on patients with HER2-positive metastatic breast cancer after prior treatment with T-DM1 (n = 184). After 11.1 months median follow-up, 60.9% (95% CI: 53.4–68.0) of the patients reported a response to the therapy [95]. A more recent update of the study with an increased duration of follow-up, from 11.1 to 20.5 months, reported a 61.4% overall response rate (ORR) with a median duration of response (DOR) of 20.8 months [31]. The median PFS was reported to be 19.4 months (95% CI: 14.1–NE) and the median OS was 24.6 months [31]. During the study, the most noted high grade adverse events in T-DXd treated group were, decreased neutrophil count, anemia, and nausea. Additionally, some patients treated with T-DXd presented with interstitial lung disease [31,95]

T-DXd was recently approved in the US for patients with HER2-positive metastatic breast cancer treated with a prior anti-HER2 based regimen [160]. This approved extension in the indication was based on positive results from the phase III clinical trial, DESTINY-BREAST03 [154,160]. The trial investigated the effect of T-DXd vs. T-DM1 in HER2-positive patients with metastatic breast cancer previously treated with a trastuzumab and taxane-based therapy (n = 524) [154]. Results from the trial showed that T-DXd improved PFS (75.8% vs. 34.1%; HR 0.28; 95% CI: 0.22–0.37; *p* < 0.001) and had an ORR of 79.7% vs. 34.2% in the T-DM1 treated cohort [154]. Moreover, it was observed that patients treated with T-DXd had a higher incidence of high-grade drug-related adverse events (45.1% vs. 39.8%) and had a higher occurrence rate of interstitial lung disease or pneumonitis (10.5% vs. 1.9%) [154].

Most recently, T-DXd received FDA approval for the treatment of adult patients with unresectable, or metastatic HER2-low breast cancer, after treatment with prior chemotherapy or after disease recurrence within six months of completing adjuvant chemotherapy [155]. The approval is based on the results from the DESTINY-Breast04 Phase III trial where T-DXd reduced the risk of disease progression or death by 50% versus physician’s choice of chemotherapy in patients with HER2-low metastatic breast cancer with hormone receptor (HR)-positive disease or HR-negative disease (n = 557) [34]. Results from the trial showed that T-DXd improved PFS (9.9 versus 5.1 months; HR 0.50; 95% CI: 0.40–0.63; *p* < 0.0001) and had an OS of 23.4 months vs. 16.8 months in patients treated with chemotherapy [34]. Moreover, grade 3 or higher adverse events occurred in 52.6% of the patients receiving T-DXd and in 67.4% of the physician’s choice of chemotherapy cohort [34].

Currently, there are ongoing clinical trials, phase Ib/II DESTINY-BREAST07 (NCT04538742) and phase Ib DESTINY-BREAST08 (NCT04556773), that are investigating the anti-tumor activity of T-DXd in combination with other therapies in patients with HER2-positive metastatic breast cancer or metastatic HER2-low advanced or metastatic Breast Cancer, respectively. The trials are still ongoing and no results have been posted yet.

### 3.5. Toxicity Profiles of FDA Approved Anti-HER2 ADCs

Clinical trials for both T-DM1 and T-DXd report that both ADCs are generally well tolerated and are mainly associated with low incidences of AE. However, there are clinical concerns regarding T-DM1-induced high-grade thrombocytopenia and T-DXd-induced high-grade interstitial lung disease [19,30,31,34,93,95,96,154]. T-DM1-induced thrombocytopenia has been regarded as a primary dose limiting or treatment terminating factor [161]. The main mechanism of T-DM1-induced thrombocytopenia is reported to be off-target uptake of T-DM1 by megakaryocytes, either through receptor binding or pinocytosis [162,163,164]. It has been suggested that structural modifications of T-DM1 could potentially improve the toxicity profile of the ADC [161].

Interstitial lung disease is an AE associated with T-DXd and, according to the FDA label, is regarded as a treatment terminating factor in patients with grade 2 or higher interstitial lung disease [31,165]. The mechanism of T-DXd related lung injury is suggested to be an off-target uptake of T-DXd into alveolar macrophages presented with features of diffuse lymphocytic infiltrates and slight fibrosis [166]. Additional studied are required to further investigate the basis behind this off-target uptake as it is not yet fully understood [157,166].

### 3.6. Anti-HER2 ADCs Undergoing Development

Multiple novel anti-HER2 ADCs are currently under development. While some ADCs are being investigated in ongoing clinical trials (Table 2), many others are being developed for further improvements in the efficacy of the ADC while eliminating any related AE. Recently, the FDA accepted for review a biological license application (BLA) for vic-trastuzumab duocarmazine (SYD985) for the treatment of advanced HER2-positive breast cancer [167]. This was based on significant results from the phase III trial, TULIP, favoring vic-trastuzumab duocarmazine over physicians’ choice of therapy in patients with pretreated locally advanced or metastatic HER2-positive breast cancer [29]. In addition, a phase I/II study, SBT6050-201 (NCT05091528), was recently announced for SBT6050, which is currently being evaluated in a phase I/Ib ongoing trial in patients with advanced HER2-overexpressing solid tumors [110].

Multiple novel ADCs designed with different technologies have also shown encouraging results. Disitamab vedotin (RC48), utilizing hertuzumab anti-HER2 antibody, was recently granted conditional approval in China for the treatment of locally advanced or metastatic HER2-overexpressing gastric cancer [168]. In the US, however, RC48 is still undergoing phase III clinical trial in patients with locally advanced or metastatic HER2-overexpressing gastric cancer (NCT04714190) [169]. ZW49 is another anti-HER2 ADC that utilizes a biparatopic antibody that recognizes the binding domains of both trastuzumab and pertuzumab [113]. ZW49 showed encouraging results in preclinical studies [113,170] and is ongoing phase I clinical trial (NCT03821233) [113]. Lastly, a pertuzumab-based ADC has recently been engineered to have less affinity for HER2 at acidic endosomal pH leading to significant improvements in lysosomal delivery and cytotoxicity when tested against HER2-low-expressing xenograft models [171]. The engineered pertuzumab variants are expected to enter clinical trials in patients with HER2-overexpressing solid tumors [171].

## 4. Conclusions

The emergence of ADCs in the last decade has revolutionized the management of HER2-positive breast cancers. The unique molecular structures of ADCs harness the specificity of HER2-targeting antibodies in guiding cytotoxic payloads to HER2-overexpressing tumors. The distinctive pharmacokinetic and pharmacodynamic properties of anti-HER2 ADCs offer solutions for high-risk, heavily pretreated patients and offer the potential to overcome limitations of HER2 resistance; they also spotlight the increased response to HER2 targeted therapy. T-DXd and T-DM1 have shown the path to transformed treatment options for HER2-positive patients and will continue to do so with ongoing clinical trials. A plenitude of growing anti-HER2 ADCs are currently in their preclinical and early clinical phases and are next in line to contribute to the growth of this field of oncology. Although one mechanism of action of ADCs, internalization and intracellular processing, has been established, there remain questions regarding other processing mechanisms and organ-injury-specific mechanisms that need to be answered to be able to safely expand the use of anti-HER2 ADCs to a wider range of patients.

## Figures and Tables

**Table 1:** Mechanisms of action of anti-HER2 agents.

Drug	Drug Type	Mechanism of Action
Trastuzumab (Herceptin^®^)	Monoclonal Antibody (mAb)	Inhibition of HER2 shedding [77]. Inhibition of PI3K/AKT pathway [78,79]. Antibody-dependent cellular cytotoxicity (ADCC) [80]. Inhibition of tumor angiogenesis [81].
Pertuzumab (PERJETA^®^)	Monoclonal Antibody (mAb)	Inhibition of HER2 heterodimerization [73]. Antibody-dependent cellular cytotoxicity (ADCC) [82].
Lapatinib (TYKERB^®^)	Small molecule tyrosine kinase inhibitor	Inhibition of intrinsic tyrosine kinase activity [83]. Prevention of downstream activation signal [84].
Neratinib (Nerlynx^®^)	Irreversible pan-erbB tyrosine kinase inhibitor	Irreversible inhibition of intrinsic tyrosine kinase activity [85].

**Table 2:** Anti-HER2 ADCs in clinical trials or on the market.

ADC Name	Indication	Trial ID	Phase	Reference
Fam-trastuzumab deruxtecan-nxki	Metastatic breast cancer	-	Approved	[93,94,95]
Ado-trastuzumab emtansine	Metastatic breast cancer	-	Approved	[19,96]
Disitamab vedotin	Metastatic gastric cancer	NCT04714190	Phase III/Approved in China	[97,98,99,100]
Vic-trastuzumab duocarmazine	Metastatic breast cancer	NCT03262935	FDA accepted BLA ^1^/Phase III	[29,101]
ZRC-3256	Metastatic breast cancer	CTRI/2018/07/014881	Phase III	[102]
TAA013	Metastatic breast cancer	CTR20200806	Phase III	[103]
ARX788	Metastatic breast cancer/gastric cancer	CTR20201708	Phase II/III	[104]
MRG002	Metastatic breast cancer	NCT04492488	Phase II	[105]
DP303c	Gastric Cancer	NCT04146610	Phase II	[106]
BDC-1001	Metastatic breast cancer/gastric cancer	NCT04278144	Phase I/II	[107]
A166	Metastatic breast cancer	NCT03602079	Phase I/II	[108,109]
SBT6050	Advanced solid tumors	NCT05091528	Phase I/II	[110]
SHR-A1811	Advanced solid tumors	NCT04446260	Phase I/II	
SHR-A1201	Metastatic breast cancer	CTR20191558	Phase I/II	
MT-5111	Advanced solid tumors	NCT04029922	Phase I	[111]
ALT-P7	Metastatic breast cancer	NCT03281824	Phase I	[112]
ZW49	Metastatic breast cancer	NCT03821233	Phase I	[113]
FS-1502	Breast Cancer	NCT03944499	Phase I	
BI-CON-02	Metastatic breast cancer	NCT03062007	Phase I	
DX126-262	Breast/gastric cancer	CTR20191224	Phase I	
HS630	Breast Cancer	CTR20181755	Phase I	
B003	Metastatic breast cancer	NCT03953833	Phase I	
GQ1001	Advanced solid tumors	NCT04450732	Phase I	

^1^ accepted for review a biologics license application (BLA).

**Table 3:** Summary of Phase III clinical trials that led to FDA approval of T-DM1.

**Trial**	**EMILIA** (HER2-positive advanced breast cancer previously treated with trastuzumab and a taxane)			
**Groups**	Experimental Therapy		Control Arm	
**Treatment**	T-DM1		Lapatinib + capecitabine	
**Sample size**	*n* = 495		*n* = 496	
**Endpoint**	**Overall** **Survival**	30.9 months	**Overall** **Survival**	25.1 months
	**Progression-free** **Survival**	9.6 months	**Progression-free** **Survival**	6.4 months
	**Grade** **≥ 3** **Adverse Events**	48%	**Grade** **≥ 3** **Adverse Events**	60%
**Trial**	**TH3RESA** (HER2-positive advanced breast cancer previously treated with both trastuzumab and lapatinib in the advanced setting and a taxane in any setting)			
**Groups**	Experimental Therapy		Control Arm	
**Treatment**	T-DM1		Physician’s Choice ^1^	
**Sample size**	*n* = 404		*n* = 198	
**Endpoint**	**Overall** **Survival**	22.7 months	**Overall** **Survival**	15.8 months
	**Progression-free** **Survival**	6.2 months	**Progression-free** **Survival**	3.3 months
	**Grade** **≥ 3** **adverse events**	40%	**Grade** **≥ 3** **adverse events**	47%
	**Treatment exposure-adjusted rate of grade** **≥ 3** **Adverse Events**	123.6/100 patient-years	**Treatment exposure-adjusted rate of grade** **≥ 3** **Adverse Events**	278.4/100 patient-years
**Trial**	**KATHERINE** (HER2-positive early breast cancer with residual invasive disease at surgery after neoadjuvant therapy with trastuzumab and a taxane)			
**Groups**	Experimental Therapy		Control Arm	
**Treatment**	T-DM1		Trastuzumab	
**Sample size**	*n* = 743		*n* = 743	
**Endpoint**	**Invasive** **disease-free survival**	87.8%	**Invasive** **disease-free survival**	77.8%
	**Freedom from distant** **recurrence**	89.5%	**Freedom from distant** **recurrence**	83.7%
	**Overall** **Survival**	94.3%	**Overall** **Survival**	92.5%
	**Grade** **≥ 3** **Adverse Events**	15.4%	**Grade** **≥ 3** **Adverse Events**	25.7%

^1^ Physician’s choice of therapy included chemotherapy, hormonal therapy, and anti-HER2 therapy.

**Table 4:** Summary of clinical trials that led to FDA approval of T-DXd.

**Trial**	**DESTINY-BREAST01** (Trastuzumab Deruxtecan in Metastatic HER2-Positive Breast Cancer Previously Treated with T-DM1)	
**Group**	Experimental Therapy (intention to treat)	
**Treatment**	T-DXd (5.4 mg/Kg)	
**Sample Size**	*n* = 184	
**Median Progression-Free Survival**	16.4 months	
**Overall Response**	60.9%	
**Drug-related Grade** **≥ 3** **adverse events**	57.1%	
**Trial**	**DESTINY-BREAST03 (Phase III Clinical Trial)** (Trastuzumab Deruxtecan versus Trastuzumab Emtansine for Breast Cancer)	
**Groups**	Experimental Therapy	Control Arm
**Treatment**	T-DXd	T-DM1
**Sample Size**	*n* = 261	*n* = 263
**Progression-Free Survival at 12 Months**	75.8%	34.1%
**Overall Response**	79.7%	34.2%
**Drug-related Grade** **≥ 3** **Adverse events**	45.1%	39.8%
**Trial**	**DESTINY-BREAST04** (Trastuzumab Deruxtecan in Previously Treated HER2-Low Advanced Breast Cancer)	
**Groups**	Experimental Therapy	Control Arm
**Treatment**	T-DXd	Physician’s Choice ^1^
**Sample size**	*n* = 373	*n* = 184
**Median Progression-Free Survival**	9.9 months	5.1 months
**Overall Survival**	23.4 months	16.8 months
**Drug-related Grade** **≥ 3** **adverse events**	52.6%	67.4%

^1^ Physician’s choice of therapy included: capecitabine, eribulin, gemcitabine, paclitaxel, or nab-paclitaxel.

## Data Availability

Not applicable.
